# Decoding Peroxidase Gene Function in Heat Stress Adaptation of *Tetranychus urticae*: Unraveling Molecular Mechanisms of Short-Term Thermal Tolerance

**DOI:** 10.3390/antiox14050562

**Published:** 2025-05-08

**Authors:** Yaonian Chen, Yuan Liu, Rangjun Wang, Pengcheng Nie, Bin Wei, Rasha S. Abdel-Fattah, Suqin Shang, Youssef Dewer

**Affiliations:** 1 Technique College of Agriculture and Forestry, Longnan Normal University, Longnan 742500, China; chenyn0935@163.com (Y.C.); 18706760096@163.com (Y.L.); wangrjlnsf@163.com (R.W.); 2 Biocontrol Engineering Laboratory of Crop Diseases and Pests of Gansu Province, College of Plant Protection, Gansu Agricultural University, Lanzhou 730070, China; niepcgs@163.com (P.N.); gsndwb@126.com (B.W.); 3 Scale Insects and Mealybugs Department, Plant Protection Research Institute, Agricultural Research Center, 7 Nadi El-Seid Street, Dokki, Giza 12618, Egypt; roshynado@yahoo.com; 4 Phytotoxicity Research Department, Central Agricultural Pesticide Laboratory, Agricultural Research Center, 7 Nadi El-Seid Street, Dokki, Giza 12618, Egypt

**Keywords:** *Tetranychus urticae*, peroxidase, short-term heat stress, bioinformatics analysis, gene expression pattern

## Abstract

*Tetranychus urticae* (Acari: Tetranychidae) is a widely distributed agricultural pest, and it possesses an exceptional capacity to withstand or adapt to short-term heat stress. To investigate the molecular mechanisms underlying this heat tolerance, using both transcriptome and whole-genome data, we identified six distinct *POD* genes in *T. urticae* and characterized their physicochemical properties and structural features. Real-time quantitative PCR (RT-qPCR) was utilized to analyze the expression profiles of these genes under short-term heat stress. Our results show that *T. urticae* mitigates heat-induced oxidative stress through the upregulation of *POD* gene expression, highlighting the critical role of these genes in the mite’s adaptive response to thermal stress. These findings contribute to a deeper understanding of the molecular pathways that enable *T. urticae* to survive in fluctuating thermal environments, which is increasingly relevant in the context of global climate change. Furthermore, this study provides a foundation for future research utilizing RNA interference (RNAi) technology to further investigate the functional roles of these *POD* genes and their potential as targets for pest control strategies.

## 1. Introduction

*Tetranychus urticae* is a major agricultural pest that causes significant damage to a wide range of crops, vegetables, flowers, fruit trees, and legumes [1]. *T. urticae* typically employs piercing-sucking mouthparts to feed on above-ground plant parts, and it disseminates via webbing and crawling between neighboring plants, causing substantial losses to agroforestry worldwide [2,3]. As global warming and the widespread use of cultivation facilities, high temperatures provide favorable conditions for the rapid population expansion of *T. urticae*, exacerbating its impact. Heat stress not only accelerates reproduction, particularly in females, but also increases egg production, thereby enhancing the mite’s capacity to infest crops [4,5]. The ability of *T. urticae* to adapt to elevated temperatures presents a growing challenge for pest management strategies, leading to more severe plant damage.

As poikilothermic organisms, mites, including *T. urticae*, are highly sensitive to environmental temperature fluctuations and can rapidly adjust their body temperature within a limited range [6]. However, prolonged exposure to elevated temperatures can overwhelm their cellular defenses, triggering the overproduction of reactive oxygen species (ROS). This imbalance between ROS generation and detoxification processes leads to oxidative stress, causing irreversible cellular damage and, ultimately, death [7,8,9]. For instance, under heat stress conditions, *Phytoseiulus persimilis* will enter a comatose state at 41.1 °C, while *T. urticae* will only do so at 48.7 °C [10]. This phenomenon has also been observed in *Tetranychus pacificus* and *Galendromus occidentalis* [11,12,13]. Furthermore, the mortality rate of *Neoseiulus californicus* in the developmental stage is very high at 35 °C, and their eggs cannot hatch when the temperature rises to 37.5 °C [14]. To counteract oxidative stress, organisms employ antioxidant enzymes, such as superoxide dismutase (SOD), catalase (CAT), and peroxidase (POD), which work synergistically to maintain a low, dynamic equilibrium of ROS [15,16]. Among these enzymes, POD is particularly critical, as it catalyzes the reduction of hydrogen peroxide (H_2_O_2_), a key ROS, into water and oxygen. This reaction helps neutralize excess H_2_O_2_ and other toxic metabolic byproducts, such as phenols, amines, and aldehydes [17].

As an oxidoreductase, POD plays a crucial role in the antioxidant defense system by catalyzing the decomposition of H_2_O_2_ and contributing to the detoxification of ROS. Notably, while both POD and CAT can decompose H_2_O_2_ into water and oxygen, POD is effective at lower cellular concentrations compared to CAT, which requires a higher concentration to achieve the same reaction [18].

Studies have demonstrated that *POD* activity and *POD* gene expression are significantly altered in response to heat stress in various insect and mite species. For example, *POD* activity in *Aphelinus asychis* [19], *Neoseiulus barkeri* [20], *Mononychellus mcgregori* [21], and *Propylaea japonica* [22] is strongly influenced by heat stress. In *Myzus persicae*, *POD* gene expression increased up to 8.1 times under heat stress compared to control conditions [23]. In *Frankliniella occidentalis*, *POD* expression was significantly elevated at 33, 37, and 39 °C relative to control conditions [24], and in *N. barkeri*, *POD* gene expression peaked after 4 h of exposure to 40 °C heat stress [25]. In addition, the activity of peroxidase of *T. urticae* at different temperatures (36 °C, 39 °C, 42 °C, and 45 °C) and different stress times (2 h, 4 h, and 6 h) was investigated in the previous stage of our group. The results of this investigation revealed that the activity of *POD* was significantly enhanced at all temperatures and stress times compared with the 25 °C control group. The lowest *POD* activity of 391.96 U/mg protein was observed at 6 h of exposure to 45 °C, whereas the highest *POD* activity of 1289.02 U/mg protein was observed at 39 °C for 4 h of stress [26].

To further explore the heat tolerance mechanisms in *T. urticae*, we conducted a comprehensive transcriptomic analysis under short-term heat stress conditions, using 25 °C as the control temperature. The resulting transcriptomic dataset was uploaded to the NCBI database for further exploration. To gain a deeper understanding of *POD*’s role under heat stress, we identified and cloned six *POD* genes from the transcriptomic data and the published genome of *T. urticae* from 2011 [27]. Bioinformatics analysis was then used to characterize the sequence features, genetic structure, and physicochemical properties of these genes. Finally, real-time quantitative PCR (RT-qPCR) was employed to analyze the expression patterns of these *POD* genes under short-term heat stress conditions.

## 2. Materials and Methods

### 2.1. Mite Colony

The *Tetranychus urticae* population used in this study was originally collected from Xinglong Mountain, China, in May 2012. For over 40 generations, the colony has been maintained in a temperature-controlled environment at Gansu Agricultural University, Lanzhou, Gansu Province, thus ensuring its population homogeneity and the absence of contamination by other species or impurities. The mites were reared on bean leaves (*Phaseolus vulgaris* L.) under acaricide-free conditions, with environmental parameters set to 25 ± 1 °C, 60 ± 5% relative humidity (RH), and a 16 h light/8 h dark photoperiod (L16:D8).

### 2.2. Selection of POD Genes

By integrating transcriptome data (Accession number: PRJNA1073827) and whole genome data (Accession number: GCA_000239435.1) from the NCBI database, the peroxidase (*POD*) genes selected for this study were identified. Gene selection was guided by functional annotations from the transcriptomic dataset, which enabled the precise identification of *POD* genes for further analysis.

### 2.3. Cloning the CDSs of POD Genes

The Open Reading Frame Finder (ORF Finder) program available on the NCBI website (https://www.ncbi.nlm.nih.gov/orffinder/ (accessed on 24 July 2024)) was used to find the coding sequences (CDS) of the *POD* genes obtained by screening transcriptome datasets. Specific primers for cloning the *POD* genes in *T. urticae* were designed using the Primer3 Input tool (https://bioinfo.ut.ee/primer3/ (accessed on 25 July 2024)) (Table S1). Three hundred healthy female adult *T. urticae* were selected for total RNA extraction using RNAiso Plus reagent (Takara, Dalian, China). RNA quality was assessed using a nanophotometer (GE Healthcare, Wiesbaden, Germany), and samples that met the quality standards were stored at −80 °C for future use. First-strand cDNA synthesis was performed using the PrimeScript II 1st Strand cDNA Synthesis Kit (Takara, Dalian, China), with the extracted RNA as the template. PCR amplification of the cDNA was subsequently carried out using PrimeSTAR Max DNA Polymerase (Takara, Dalian, China) (Tables S2 and S3), and the amplified products were purified using the TaKaRa MiniBEST Agarose Gel DNA Extraction Kit Ver. 4.0 (Takara, Dalian, China). The purified PCR products were then ligated into the pLB-T vector (TIANGEN, Beijing, China) and transformed into *Escherichia coli* TOP10 cells (TIANGEN, Beijing, China) for propagation using the thermal shock method. Positive clones were selected, and plasmid sequencing was performed by Sangon Biotech Co., Ltd. (Shanghai, China). The obtained CDS sequences were compared with the whole genome sequence of *T. urticae* [27] to verify the accuracy of the results.

The selection of healthy adult female *T. urticae* mites: During the collection of *T. urticae* for experimental purposes, it was possible to clearly observe the gender and developmental stage of the target mites through the use of a microscope. If the mites exhibited overt signs of natural activity responses upon being touching with a small brush, they were considered healthy and active.

### 2.4. Identification and Phylogenetic Analysis of POD Proteins

The protein sequence is obtained through translation of the coding sequence (CDS) region of the gene sequence. To verify the accuracy of the sequencing of the clone results, the ORF Finder (https://www.ncbi.nlm.nih.gov/orffinder/ (accessed on 15 August 2024)) was used to determine the open reading frame (ORF) of the cloned gene sequences, after which translation was conducted to obtain the corresponding protein sequence on the same website. The protein sequences obtained were compared with the whole genome sequence of *T. urticae* [27]. The identification of functionally conserved domains was performed using the Protein Families Database (Pfam) (https://www.ebi.ac.uk/interpro/search/sequence/ (accessed on 20 August 2024)) and the Conserved Domain Search Service (CD Search) from the NCBI website (https://www.ncbi.nlm.nih.gov/Structure/cdd/wrpsb.cgi (accessed on 21 August 2024)). Based on the identified domains, the proteins were classified accordingly. The conserved motifs of the POD proteins were predicted using the MEME tool (https://meme-suite.org/meme/tools/meme (accessed on 21 August 2024)), and the resulting motifs were visualized using TBtools (Toolbox for Biologists) v0.6735 software. Additionally, POD protein sequences from other insect and mite species were retrieved from the NCBI database. A phylogenetic tree was constructed using the neighbor-joining method in MEGA7 software, with 1000 bootstrap replicates, combining the *T. urticae* POD protein sequences for comparative analysis.

### 2.5. Bioinformatic Analysis of POD Proteins

To investigate the physicochemical properties of the POD proteins from *T. urticae*, the ExPASy-ProtParam online tool (http://web.expasy.org/protparam (accessed on 21 August 2024)) was used to perform the necessary calculations. Furthermore, the NetNGlyc 1.0 server (https://services.healthtech.dtu.dk/services/NetNGlyc-1.0/ (accessed on 21 August 2024)) and the NetPhos 3.1 server (https://services.healthtech.dtu.dk/services/NetPhos-3.1/ (accessed on 21 August 2024)) were utilized to predict potential N-glycosylation and phosphorylation sites, respectively. The TMHMM 2.0 server (https://services.healthtech.dtu.dk/services/TMHMM-2.0/ (accessed on 22 August 2024)) was employed to predict the transmembrane topology of the POD proteins. Additionally, the ExPASy-Protscale tool (http://web.expasy.org/protscale (accessed on 22 August 2024)) was used to analyze and predict the hydrophilicity profiles of the POD proteins. To predict the presence of signal peptides and determine the subcellular localization of the target proteins, SignalP-6.0 (https://services.healthtech.dtu.dk/services/SignalP-6.0/ (accessed on 22 August 2024)) and WOLF PSORT (https://wolfpsort.hgc.jp/ (accessed on 22 August 2024)) were used, respectively.

For a more detailed structural understanding of the POD proteins in *T. urticae*, the secondary structure was predicted using the SOPMA tool (https://npsa.lyon.inserm.fr/cgi-bin/npsa_automat.pl?page=/NPSA/npsa_sopma.html (accessed on 23 August 2024)). The tertiary structure was predicted by means of the AlphaFold2 online tool, and the visual representations of structures were generated using the PYMOL software program [28,29], providing insights into the overall protein folding and potential functional regions.

### 2.6. Short-Term Heat Stress Treatment of T. urticae

A pre-experiment was conducted to ascertain the mortality rate of *T. urticae* under heat stress conditions. The results of this experiment indicated that the mortality of the test mites occurred when the external temperature exceeded 42 °C. Therefore, to ensure the survival of the test mites during the experiment and to simulate the field temperature, 36 °C, 39 °C, and 42 °C were selected as the experimental temperatures in the formal experiment. In the formal experiment, the infested leaves containing *T. urticae* mites were placed in the controlled climate chambers and subjected to heat stress at 36 °C, 39 °C, and 42 °C for 4 h, with mites reared at 25 °C serving as the control group. All treatments were conducted under constant humidity conditions (60 ± 5% RH). Following the heat stress exposure, 200 surviving female adult mites from each treatment were collected into 1.5 mL centrifuge tubes (pH = 7). Each treatment was replicated three times biologically. The collected samples were then stored at −80 °C until RNA extraction.

### 2.7. Analysis of POD Gene Expression

The *T. urticae* mites, collected as described in Section 2.6, were used for RNA extraction, following the protocol outlined in Section 2.3. Total RNA was reverse transcribed into complementary DNA (cDNA) using the PrimeScript™ RT Reagent Kit with gDNA Eraser (Perfect Real Time) (Takara, Dalian, China). Based on the gene sequences obtained from cloning, specific primers for RT-qPCR were designed using the Primer3 Input online tool (refer to Table S1). For normalization of gene expression levels, the α-tubulin gene (GenBank Accession: JN881327.1) [30] was selected as the internal reference gene. RT-qPCR was conducted on an ABI QuantStudio 5 Real-Time PCR System with Hieff UNICON qPCR SYBR Green Master Mix (Yeasen, Shanghai, China) (Tables S4 and S5). Three technical replicates were performed for each biological replicate. Gene expression levels were quantified using the 2^−ΔΔCt^ method [31].

### 2.8. Statistical Analysis

All statistical analyses in this study were performed using SPSS software (IBM SPSS Statistics 26, IBM Corporation, Somers, NY, USA) to compare relative gene expression levels. One-way analysis of variance (ANOVA), followed by Duncan’s multiple range test (*p* < 0.05), was used to evaluate differences among treatments. Graphs and figures were created using Origin 2018 software.

## 3. Results

### 3.1. Selection of POD Genes

**Figure 1:**
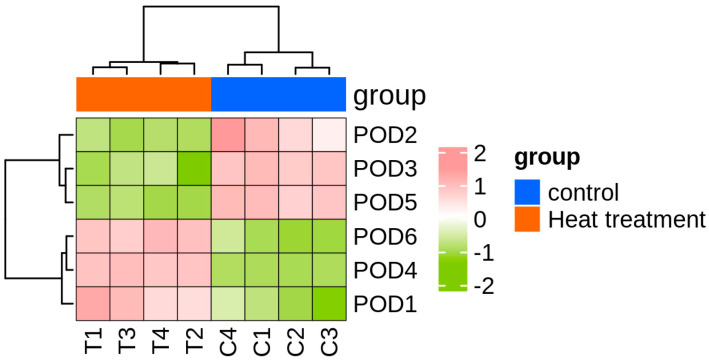
Heatmap of *TuPODs* gene expression response to short-term heat stress. C1-C4: represent four independent biological replicates of control samples; T1-T4: represent four independent biological replicates of heat-treated samples. The color scale at the bottom indicates the FPKM (fragments per kilobase of transcript per million mapped reads) values, ranging from the lowest (green) to the highest (pink).

Six peroxidase (*POD*) genes were identified based on functional annotation from transcriptome sequencing conducted on *T. urticae* exposed to high temperature (39 °C) and normal temperature (25 °C). Transcriptomic analysis indicated that the FPKM (fragments per kilobase of transcript per million mapped reads) values of *TuPOD1*, *TuPOD4*, and *TuPOD6* showed a significant increase under heat stress, whereas the expression of other *POD* genes was significantly decreased (Figure 1).

### 3.2. Verification of the Accuracy of Cloned POD Protein Sequences

In this study, six *POD* genes from *T. urticae* were successfully cloned and deposited in the NCBI database with the following accession numbers: PQ816576.1, PQ816577.1, PQ816578.1, PQ816579.1, PQ816580.1, and PQ816581.1. To verify the accuracy of the cloned sequences, a homology search was performed, revealing that the cloned POD proteins exhibited 100% identity with the corresponding sequences in the *T. urticae* reference genome available in the NCBI database (Table 1). This confirmation of sequence identity further validates the reliability of the cloned POD proteins for subsequent analyses.

### 3.3. Sequence and Phylogenetic Analysis of POD Proteins

**Figure 2:**
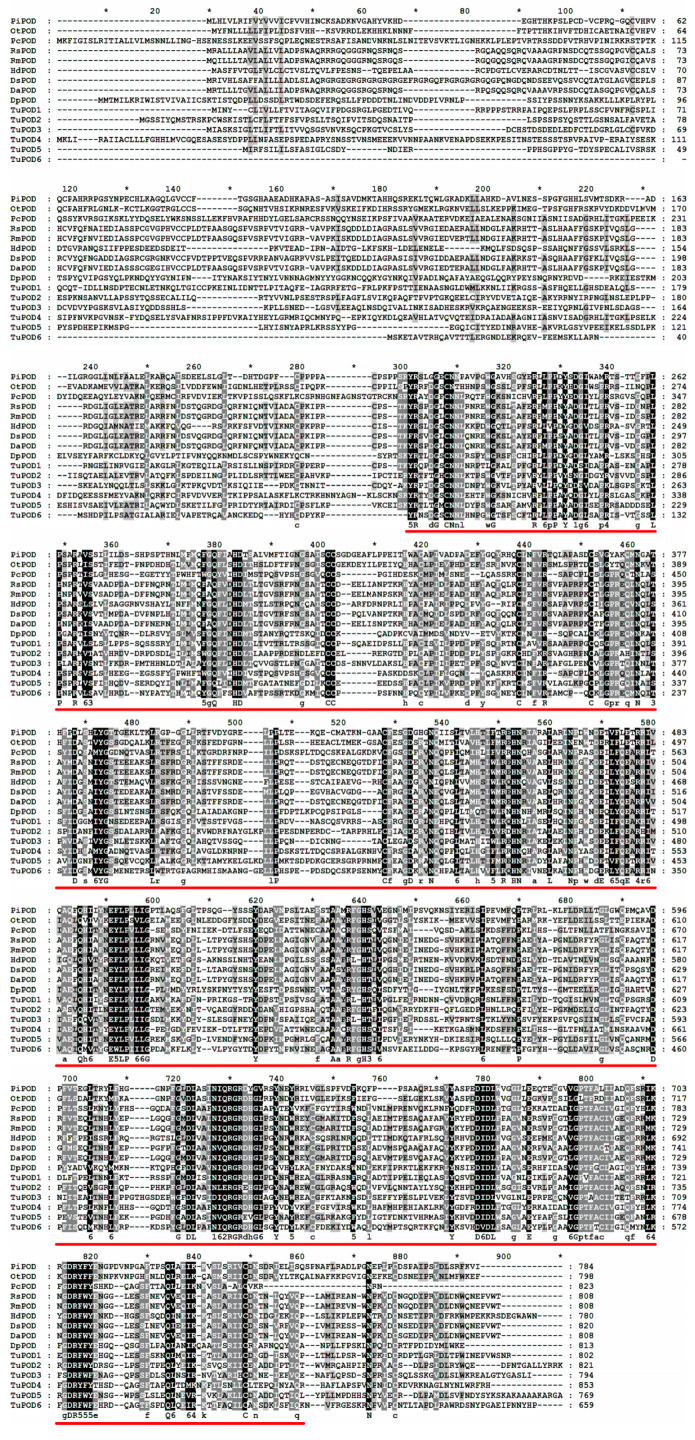
Multiple sequence alignment of peroxidase proteins of *T. urticae* with other species. (PiPOD: *Plodia interpunctella* POD; OtPOD: *Onthophagus taurus* POD; PcPOD: *Panonychus citri* POD; RsPOD: *Rhipicephalus sanguineus* POD; RmPOD: *Rhipicephalus microplus* POD; HdPOD: *Halotydeus destructor* POD; DsPOD: *Dermacentor silvarum* POD; DaPOD: *Dermacentor andersoni* POD; DpPOD: *Dermatophagoides pteronyssinus* POD). Animal heme peroxidases domain (An_peroxidase) is indicated by red underlines.

**Figure 3:**
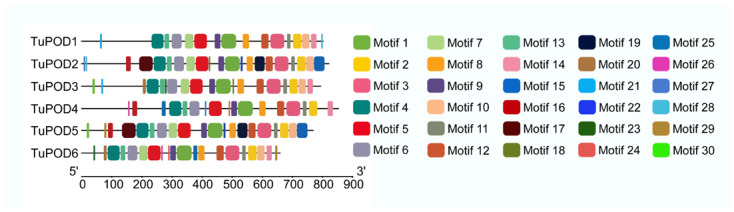
Distribution of conserved motifs in TuPODs proteins. The motif distribution of the six *T. urticae* POD proteins, highlighting the number and arrangement of conserved motifs across each protein.

**Figure 4:**
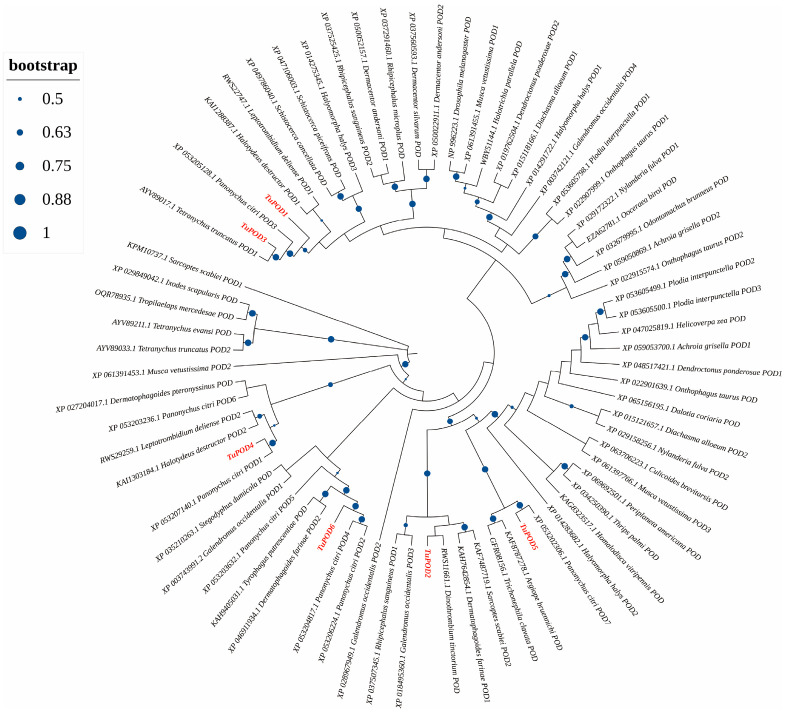
Phylogenetic analysis of TuPODs proteins. A phylogenetic tree based on the peroxidase (POD) protein sequences of *T. urticae* and related species. Target genes are indicated in red font, and the size of the blue orb represents the node reliability of the phylogenetic tree.

The functional domain analysis revealed that all six TuPOD proteins from *T. urticae* contain conserved peroxide structural domains, characteristic of animal heme peroxidases (An_peroxidase) (Figure 2). Each protein was found to possess 10 heme-binding sites and three calcium (Ca^2+^) binding sites, further confirming their classification as peroxidases (Figure S1). A total of 30 conserved motifs were identified across the six POD proteins (Figure 3), with TuPOD1 containing the fewest motifs (16), while TuPOD2 exhibited the most (21). Phylogenetic analysis of the peroxidase proteins from *T. urticae* (Figure 4) showed that TuPOD2 and TuPOD3 are most closely related to the peroxidase proteins from *Dinothrombium tinctorium* and *Tetranychus truncatus*, respectively. TuPOD4, TuPOD5, and TuPOD6 form a clade with the peroxidase proteins of *Panonychus citri*, whereas TuPOD1 is closely related to the peroxidase proteins of both *P. citri* and *T. truncatus*.

### 3.4. Physicochemical Properties and Protein Structure of TuPODs

The physicochemical properties of the six TuPOD proteins were analyzed to gain insight into their structural and functional characteristics. The molecular weights of TuPOD1, TuPOD2, TuPOD3, TuPOD4, TuPOD5, and TuPOD6 were determined to be 89.09 kDa, 92.67 kDa, 88.12 kDa, 95.91 kDa, 87.03 kDa, and 74.81 kDa, respectively. The theoretical isoelectric points (pI) varied across the six proteins: TuPOD1 (pI = 8.16), TuPOD2 (pI = 6.07), TuPOD3 (pI = 5.02), TuPOD4 (pI = 8.29), TuPOD5 (pI = 6.17), and TuPOD6 (pI = 6.88). The aliphatic indices, which are indicative of the proteins’ hydrophobicity, ranged from 67.22 to 91.33, with TuPOD1 showing the highest value (91.33) and TuPOD6 the lowest (67.22). These values provide useful information on protein quantification and characterization.

The instability indices, which predict the stability of the proteins, were calculated for all six TuPOD proteins and found to range from 40.05 to 54.34, indicating that these proteins are relatively unstable. This suggests that the TuPOD enzymes may be subject to degradation under certain conditions. Additionally, all six TuPOD proteins were classified as hydrophilic, reflecting the likely water-soluble nature of the proteins (Table 2, Figures S2–S6).

Regarding post-translational modifications, the N-glycosylation site prediction revealed variability in the number of sites across the TuPOD proteins. TuPOD1, TuPOD2, and TuPOD6 each had two N-glycosylation sites, while TuPOD3 contained 10, TuPOD4 had 4, and TuPOD5 had 1. Phosphorylation site prediction indicated a higher number of phosphorylation sites, ranging from 58 (TuPOD6) to 90 (TuPOD3 and TuPOD4). These modifications may play a key role in the regulation of protein function and activity.

**Figure 5:**
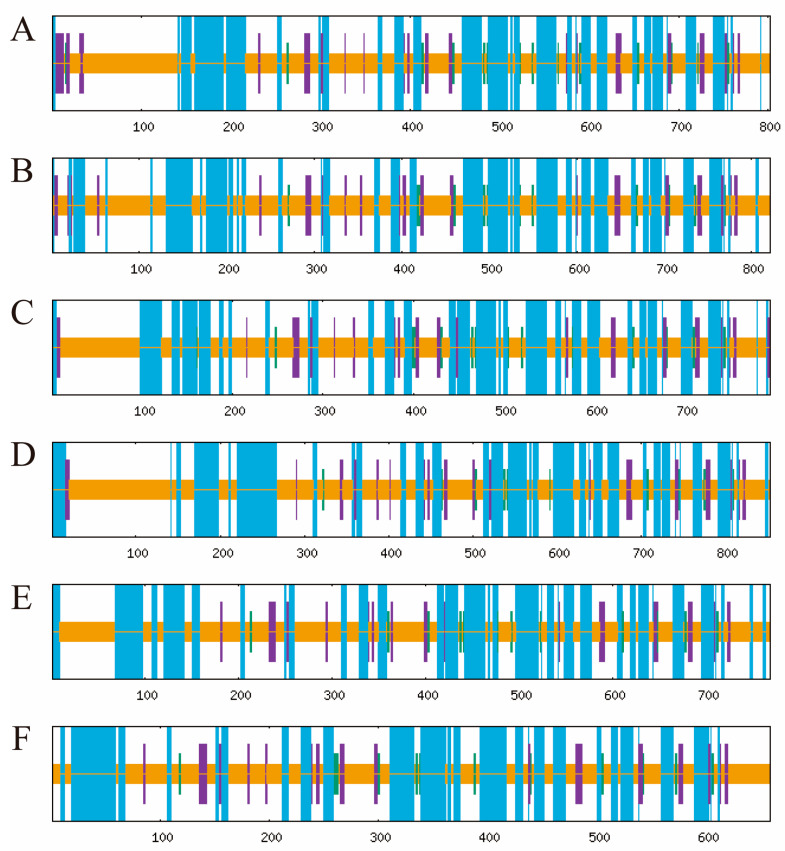
Prediction of the secondary structure of TuPOD proteins. This figure illustrates the secondary structure prediction for each of the six *T. urticae* peroxidase proteins ((**A**). TuPOD1, (**B**). TuPOD2, (**C**). TuPOD3, (**D**). TuPOD4, (**E**). TuPOD5, and (**F**). TuPOD6). Random coil is represented by orange, alpha helix by blue, extended strand by purple, and beta turn by green.

**Figure 6:**
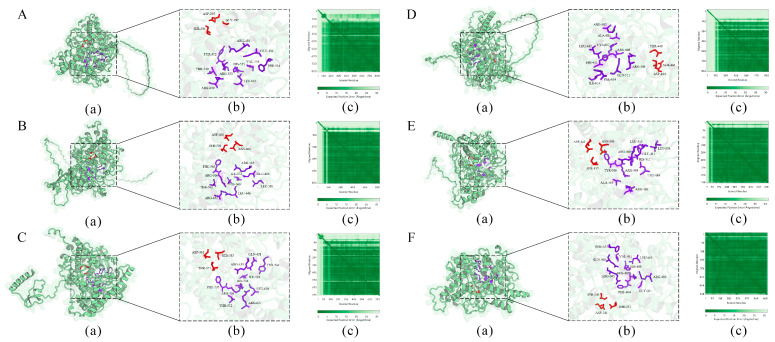
Prediction of the tertiary structure of TuPOD proteins ((**A**). TuPOD1, (**B**). TuPOD2, (**C**). TuPOD3, (**D**). TuPOD4, (**E**). TuPOD5, and (**F**). TuPOD6). (**a**) The protein structure of TuPOD proteins. (**b**) All the binding sites of TuPOD proteins. The amino acid stick structure at the calcium binding sites is shown in red, and the amino acid stick structure at the heme-binding sites is shown in purple. (**c**) Expected position error (TuPOD1-0.85, TuPOD2-0.86, TuPOD3-0.85, TuPOD4-0.80, TuPOD5-0.90, TuPOD6-0.95).

Signal peptide prediction revealed that all six TuPOD proteins, except TuPOD6, possess a Sec/SP I signal peptide, suggesting that they are likely secretory proteins. In addition, transmembrane structure predictions indicated that TuPOD1 and TuPOD2 contain a transmembrane helix region, hinting at their potential membrane association. Subcellular localization analysis confirmed that TuPOD6 is localized in the cytoplasm, whereas the other five PODs are predominantly found in the plasma membrane (Table 2, Figures S2–S6). For secondary structure predictions, the majority of the TuPODs were composed of random coils, with lesser proportions of α-helices, extended strands, and β-turns. Specifically, random coils represented the largest proportion of the structure in all six proteins, followed by α-helices, extended strands, and β-turns (Table 3, Figure 5 and Figure S7). Tertiary structure predictions further supported these findings, providing insight into the three-dimensional folding of the proteins and confirming the structural features predicted in the secondary structure analysis (Figure 6).

### 3.5. Differential Expression of TuPOD Genes Under Short-Term Heat Stress

**Figure 7:**
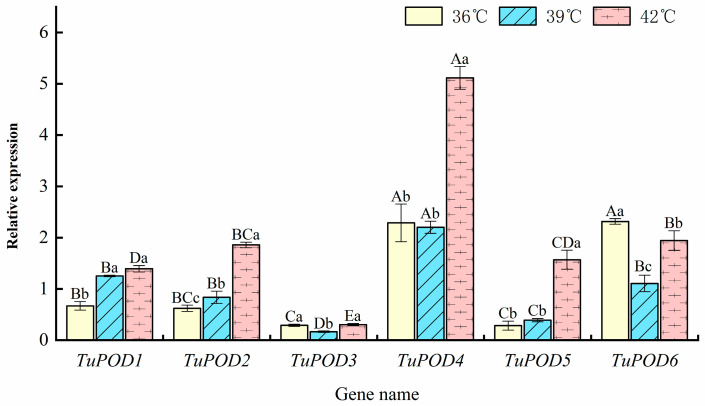
Relative expression of *TuPOD* genes under different heat stress conditions at the same duration. This figure presents the relative expression levels of the six *T. urticae* peroxidase genes under varying heat stress conditions (36 °C, 39 °C, and 42 °C) for the same duration. The expression levels are normalized to the control group (25 °C), which serves as a baseline for comparison. Data are presented as means ± SE. Uppercase letters denote significant differences (*p* < 0.05) in relative gene expression among different genes at the same temperature, while lowercase letters indicate significant differences (*p* < 0.05) in relative expression of the same gene across different temperatures, as determined by Duncan’s new multiple range test.

The expression levels of *POD* genes in *T. urticae* under heat stress were evaluated using RT-qPCR, and the results are presented in Figure 7. At 36 °C, the expression of *TuPOD4* and *TuPOD6* was upregulated by 2.28- and 2.32-fold, respectively, while the expression of *TuPOD1*, *TuPOD2*, *TuPOD3*, and *TuPOD5* was downregulated. At 39 °C, *TuPOD1*, *TuPOD4*, and *TuPOD6* expression increased by 1.25, 2.20, and 1.11-fold, respectively, whereas *TuPOD2*, *TuPOD3*, and *TuPOD5* expression was suppressed. This result was similar to the transcriptomic analysis result (Figure 1). At 42 °C, the expression of *TuPOD1*, *TuPOD2*, *TuPOD4*, *TuPOD5*, and *TuPOD6* was induced by 1.39, 1.55, 5.12, 1.57, and 1.94-fold, respectively, compared to the control group, whereas the expression of *TuPOD3* was inhibited.

Across all heat stress treatments (36 °C, 39 °C, and 42 °C for 4 h), the expression of *TuPOD1*, *TuPOD2*, and *TuPOD5* exhibited a consistent increase with rising temperatures. In contrast, the expression of *TuPOD3*, *TuPOD4*, and *TuPOD6* decreased between 36 °C and 39 °C, but subsequently increased from 39 °C to 42 °C. Notably, the expression of most *TuPOD* genes peaked at 42 °C, with the exception of *TuPOD6*, which reached its highest expression at 36 °C.

## 4. Discussion

The two-spotted spider mite, *Tetranychus urticae*, is a globally significant agricultural pest [32], and temperature is one of the key abiotic factors that influence its survival and reproduction [33]. High temperatures, in particular, can lead to substantial oxidative stress, primarily through the accumulation of reactive oxygen species (ROS). These ROS can cause cellular damage, potentially impairing vital physiological processes. Previous studies have demonstrated that *T. urticae* responds to high temperatures by modulating antioxidant enzyme activities, particularly through the involvement of superoxide dismutase (*SOD*) genes [30]. However, the role of peroxidase (*POD*) genes in the heat stress response of this mite remains underexplored. In the present study, we successfully cloned and characterized six *POD* genes from *T. urticae* and investigated their expression patterns under different heat stress conditions, providing novel insights into the molecular mechanisms of heat tolerance in this species.

In this study, we identified six TuPOD proteins, all of which belong to the animal hemoperoxidase family. Phylogenetic analysis revealed distinct evolutionary relationships among the TuPOD proteins, which could be attributed to variations in their molecular structures [34]. N-glycosylation site predictions indicated that these POD proteins may function as glycoproteins, similar to homologous proteins in other species [35,36]. Notably, a signal peptide was predicted in TuPOD1–5, suggesting that these enzymes are secretory proteins, a finding that was further validated by subcellular localization analysis [19,22,37]. These results suggest that POD proteins play an essential role in the extracellular defense against oxidative damage during heat stress.

POD enzymes work synergistically with SOD to mitigate oxidative stress by neutralizing hydrogen peroxide (H_2_O_2_) generated during the dismutation of superoxide anions. Our results indicated that the expression of five of the six *POD* genes was significantly upregulated under heat stress, which suggests that the upregulation of *POD* genes contributes to enhanced oxidative stress defense in *T. urticae*. This observation is consistent with studies in other species, such as *Aphelinus asychis* [19], *Frankliniella occidentalis* [24], *Aphidius gifuensis* [38], and *Scapharca broughtonii* [39], where up-regulation of *POD* genes expression has been observed in response to heat stress.

Interestingly, the expression patterns of *TuPOD3*, *TuPOD4*, and *TuPOD6* in female adults followed a trend of initial up-regulation followed by down-regulation. We hypothesize that the initial up-regulation of gene expression is a rapid response to produce antioxidant enzymes capable of scavenging ROS. However, as the mite’s defense system successfully mitigates oxidative damage, the expression of these genes may decrease to prevent an excess of protein accumulation, which could be energetically costly or detrimental to cellular homeostasis. This phenomenon is consistent with previous research showing that *T. urticae* exhibits higher antioxidant enzyme activity at 39 °C compared to other temperatures, and similar results have been reported by Yuan et al. (2015) [24], who proposed that organisms experiencing elevated protein production levels may suppress gene transcription to maintain physiological balance.

In contrast, the expression of *TuPOD1*, *TuPOD2*, and *TuPOD5* increased steadily with rising temperatures. This pattern is similar to the upregulation of antioxidant enzyme genes observed in *Aphelinus asychis* female adults under heat stress [19]. We speculate that high temperatures initially increase *SOD* activity to neutralize free radicals [26,29]; however, when temperatures exceed a critical threshold, *T. urticae* increases the expression of *POD* genes to enhance its antioxidant capacity and cope with the elevated ROS levels. This observation further underscores the complexity of the heat stress response, where different antioxidant enzyme genes may exhibit varied expression patterns depending on the severity of the thermal stress, as previously noted by Yuan et al. (2015) [24].

## 5. Conclusions

Our study confirms that the expression of *TuPOD* genes is modulated by heat stress in *T. urticae*, with most *POD* genes showing higher expression levels under heat stress compared to control conditions (25 °C). These findings underscore the crucial role of POD enzymes in the adaptive response of *T. urticae* to high temperatures, highlighting their potential in mitigating oxidative damage. This study not only contributes to a deeper understanding of the molecular basis of thermal stress tolerance in *T. urticae* but also provides a valuable foundation for future transcriptomic investigations and pest management strategies. The role of *POD* genes in antioxidant defense mechanisms offers important insights for understanding how this pest adapts to climate change and fluctuating environmental conditions, providing a framework for the development of more sustainable control methods in the face of global warming.

## Figures and Tables

**Table 1:** Homology comparison of TuPODs proteins. A homology comparison of the six *T. urticae* proteins with the whole genome sequences of *T. urticae* from the NCBI database.

Protein	Accession Numbers	Query Cover	E-Value	Identity
TuPOD1	XP_025017963.1 peroxidase [*Tetranychus urticae*]	100%	0.0	100.00%
TuPOD2	XP_015784100.1 peroxidase [*Tetranychus urticae*]	100%	0.0	100.00%
TuPOD3	XP_015781195.1 peroxidase [*Tetranychus urticae*]	100%	0.0	100.00%
TuPOD4	XP_015787917.1 peroxidase [*Tetranychus urticae*]	100%	0.0	100.00%
TuPOD5	XP_015785823.1 peroxidase [*Tetranychus urticae*]	100%	0.0	100.00%
TuPOD6	XP_015795696.1 peroxidase isoform X1 [*Tetranychus urticae*]	100%	0.0	100.00%
	XP_025015959.1 peroxidase isoform X2 [*Tetranychus urticae*]	100%	0.0	100.00%
	XP_015795697.1 peroxidase isoform X3 [*Tetranychus urticae*]	100%	0.0	100.00%

**Table 2:** Detailed biological information of TuPOD proteins. This table summarizes the detailed biological properties of the six peroxidase (POD) proteins identified in *T. urticae*, including their physicochemical characteristics, post-translational modifications, and subcellular localization.

Protein	aa	ORF	MW (KDa)	Isoelectric Point (pI)	Aliphatic Index	Instability Index	Hydrophilicity	N-Glycosylation Sites	Phosphorylation Sites	Signal Peptide	Transmembrane Helix	Subcellular Localization
TuPOD1	802	2409	89.09	8.16	91.33	54.34	Hydrophilic	2	78	Yes	Yes	Plasma Membrane
TuPOD2	821	2466	92.67	6.07	84.36	49.55	Hydrophilic	2	77	Yes	No	Plasma Membrane
TuPOD3	794	2385	88.12	5.02	87.81	40.05	Hydrophilic	10	90	Yes	No	Plasma Membrane
TuPOD4	853	2562	95.91	8.29	78.02	49.69	Hydrophilic	4	90	Yes	No	Plasma Membrane
TuPOD5	769	2310	87.03	6.17	82.22	44.88	Hydrophilic	1	67	Yes	No	Plasma Membrane
TuPOD6	659	1980	74.81	6.88	67.22	40.13	Hydrophilic	2	58	Yes	No	Cytoplasm

**Table 3:** Secondary structure prediction of TuPOD proteins. This table summarizes the predicted secondary structure composition of the six *T. urticae* peroxidase proteins, including the proportions of random coils, alpha helices, extended strands, and beta turns.

Protein Name	Alpha Helix	Extended Strand	Beta Turn	Random Coil
TuPOD1	274 (34.16%)	69 (8.60%)	26 (3.24%)	433 (53.99%)
TuPOD2	298 (36.30%)	60 (7.31%)	20 (2.44%)	443 (53.96%)
TuPOD3	282 (35.52%)	58 (7.30%)	22 (2.77%)	432 (54.41%)
TuPOD4	313 (36.69%)	51 (5.98%)	18 (2.11%)	471 (55.22%)
TuPOD5	289 (37.58%)	48 (6.24%)	20 (2.60%)	412 (53.58%)
TuPOD6	257 (39.00%)	50 (7.59%)	22 (3.34%)	330 (50.08%)

## Data Availability

The data presented in the study are deposited in the Science Data Bank, the data https://doi.org/10.57760/sciencedb.22345 (accessed on 19 March 2025).

## Supplementary Materials

The following supporting information can be downloaded at: https://www.mdpi.com/article/10.3390/antiox14050562/s1, Table S1: Primer sequences used in this study; Table S2: Reaction system of PCR; Table S3: Reaction conditions of PCR; Table S4: Reaction system of RT-qPCR; Table S5: Reaction conditions of RT-qPCR; Table S6: Quality control table of RNA, cDNA, and plasmid used in cloning; Table S7: Quality control table of RNA and cDNA used in RT-qPCR; Figure S1: Distribution of structural sites of *TuPODs*; Figure S2: Predicted representation of phosphorylation sites of *TuPODs*; Figure S3: Prediction of the transmembrane structure of *TuPODs*; Figure S4: Prediction of hydrophilicity of *TuPODs*; Figure S5: Signal peptide prediction of *TuPODs*; Figure S6: Predicted representation of N-glycosylation sites of *TuPODs*; Figure S7: Prediction of the secondary structure of *TuPODs*.

## References

[1] Bensoussan N., Santamaria M.E., Zhurov V., Diaz I., Grbić M., Grbić V. (2016). Plant-herbivore interaction: Dissection of the cellular pattern of *Tetranychus urticae* feeding on the host plant. Front. Plant. Sci..

[2] Assouguem A., Kara M., Ramzi A., Annemer S., Kowalczyk A., Ali E.A., Moharram B.A., Lazraq A., Farah A. (2022). Evaluation of the effect of four bioactive compounds in combination with chemical product against two spider mites *Tetranychus urticae* and *Eutetranychus orientalis* (Acari: Tetranychidae). Evid-Based Compl. Alt..

[3] Bocianowski J., Jakubowska M., Zawada D., Dobosz R. (2022). The effect of acaricide control of the two-spotted spider mite *Tetranychus urticae* Koch on the cultivation of sugar beet (*Beta vulgaris* L.) and on the size and quality of the yield. Appl. Sci..

[4] Gotoh T., Moriya D., Nachman G. (2015). Development and reproduction of five *Tetranychus* species (Acari: Tetranychidae): Do they all have the potential to become major pests?. Exp. Appl. Acarol..

[5] Thomas T., Gösta N., Bernhard S., Ida S., Andreas W. (2023). Parental exposure to heat waves improves offspring reproductive investment in *Tetranychus urticae* (Acari: Tetranychidae), but not in its predator, Phytoseiulus persimilis (Acari: Phytoseiidae). Ecol. Evol..

[6] Filazzola A., Matter S.F., MacIvor J.S. (2021). The direct and indirect effects of extreme climate events on insects. Sci. Total Environ..

[7] Stazione L., Norry F.M., Sambucetti P. (2019). Heat-hardening effects on mating success at high temperature in *Drosophila melanogaster*. J. Therm. Biol..

[8] Paaijmans K.P., Heinig R.L., Seliga R.A., Blanford J.I., Blanford S., Murdock C.C., Thomas M.B. (2013). Temperature variation makes ectotherms more sensitive to climate change. Global Chang. Biol..

[9] Xiao L., Huang L.L., He H.M., Xue F.S., Tang J.J. (2023). Life history responses of the small brown planthopper *Laodelphax striatellus* to temperature change. J. Therm. Biol..

[10] Coombs M.R., Bale J.S. (2013). Comparison of thermal activity thresholds of the spider mite predators *Phytoseiulus macropilis* and *Phytoseiulus persimilis* (Acari: Phytoseiidae). Exp. Appl. Acarol..

[11] Stavrinides M.C., Daane K.M., Lampinen B.D., Mills N.J. (2010). Plant water stress, leaf temperature, and spider mite (Acari: Tetranychidae) outbreaks in California vineyards. Environ. Entomol..

[12] Stavrinides M.C., Lara J.R., Mills N.J. (2010). Comparative influence of temperature on development and biological control of two common vineyard pests (Acari: Tetranychidae). Biol. Control.

[13] Stavrinides M.C., Mills N.J. (2011). Influence of temperature on the reproductive and demographic parameters of two spider mite pests of vineyards and their natural predator. BioControl.

[14] Gotoh T., Yamaguchi K., Mori K. (2004). Effect of temperature on life history of the predatory mite *Amblyseius (Neoseiulus) californicus* (Acari: Phytoseiidae). Exp. Appl. Acarol..

[15] Kaur M., Chadha P., Kaur S., Kaur A. (2020). Effect of *Aspergillus flavus* on lipid peroxidation and activity of antioxidant enzymes in midgut tissue of *Spodoptera litura* larvae. Arch. Phytopathol. Plant..

[16] Dampc J., Kula-Maximenko M., Molon M., Durak R. (2020). Enzymatic defense response of apple aphid *Aphis pomi* to increased temperature. Insects.

[17] Foyer C.H., Shigeoka S. (2011). Understanding oxidative stress and antioxidant functions to enhance photosynthesis. Plant Physiol..

[18] Hughes A.L. (2012). Evolution of the heme peroxidases of Culicidae (Diptera). Psyche-J. Entomol..

[19] Liu X., Fu Z.X., Kang Z.W., Li H., Liu T.X., Wang D. (2022). Identification and characterization of antioxidant enzyme genes in parasitoid *Aphelinus asychis* (Hymenoptera: Aphelinidae) and expression profiling analysis under temperature stress. Insects.

[20] Li W.Z., Zhu T., Zhou J.J., Shang S.Q. (2022). Effects of short-term heat stress on the activity of three antioxidant enzymes of predatory mite *Neoseiulus barkeri* (acari, phytoseiidae). Front. Physiol..

[21] Lu F.P., Chen Q., Chen Z.S., Lu H., Xu X.L., Jing F.L. (2014). Effects of heat stress on development, reproduction and activities of protective enzymes in *Mononychellus mcgregori*. Exp. Appl. Acarol..

[22] Zhang S.Z., Fu W.Y., Li N., Zhang F., Liu T.X. (2015). Antioxidant responses of *Propylaea japonica* (Coleoptera: Coccinellidae) exposed to high temperature stress. J. Insect Physiol..

[23] Khurshid A., Inayat R., Tamkeen A., UIHaq I., Li C.C., Boamah S., Zhou J.J., Liu C.Z. (2021). Antioxidant enzymes and heat-shock protein genes of green peach aphid (*Myzus persicae*) under short-time heat stress. Front. Physiol..

[24] Yuan J.W., Zheng Y.T., Chang Y.W., Bai J., Qin J., Du Y.Z. (2021). Differential regulation of antioxidant enzymes in *Frankliniella occidentalis* (Thysanoptera: Thripidae) exposed to thermal stress. PeerJ.

[25] Zhu T., Li W.Z., Xue H., Dong S.B., Wang J.H., Shang S.Q., Dewer Y. (2023). Selection, identification, and transcript expression analysis of antioxidant enzyme genes in *Neoseiulus barkeri* after short-term heat stress. Antioxidants.

[26] Nie P.C., Yang R.L., Zhou J.J., Dewer Y., Shang S.Q. (2023). Elucidating the effect of temperature stress on the protein content, total antioxidant capacity, and antioxidant enzyme activities in *Tetranychus urticae* (Acari: Tetranychidae). Insects.

[27] Grbić M., Van Leeuwen T., Clark R.M., Rombauts S., Rouzé P., Grbić V., Osborne E.J., Dermauw W., Cao T.N.P., Ortego F. (2011). The genome of Tetranychus urticae reveals herbivorous pest adaptations. Nature.

[28] Li W.Z., Kang W.J., Zhou J.J., Shang S.Q., Shi S.L. (2023). The antennal transcriptome analysis and characterizations of odorant-binding proteins in *Megachile saussurei* (Hymenoptera, Megachilidae). BMC Genom..

[29] Jumper J., Evans R., Pritzel A., Green T., Figurnov M., Ronneberger O., Tunyasuvunakool K., Bates R., Žídek A., Potapenko A. (2021). Highly accurate protein structure prediction with AlphaFold. Nature.

[30] Wei B., Nie P.C., Liu Y., Hou N.Y., Shi F.Y., Shao J.W., Gao Y.X., Shang S.Q., Dewer Y. (2024). Molecular identification and characterization of the superoxide dismutase (SOD) gene family in *Tetranychus urticae* (Acari: Tetranychidae) and the role of *TuSOD2* gene under short-term heat stress. Int. J. Biol. Macromol..

[31] Livak K.J., Schmittgen T.D. (2001). Analysis of relative gene expression data using real-time quantitative PCR and the 2^-ΔΔCT^ method. Methods.

[32] Adesanya A.W., Lavine M.D., Moural T.W., Lavine L.C., Zhu F., Walsh D.B. (2021). Mechanisms and management of acaricide resistance for *Tetranychus urticae* in agroecosystems. J. Pest Sci..

[33] González-Tokman D., Córdoba-Aguilar A., Dáttilo W., Lira-Noriega A., Sánchez-Guillén R.A., Villalobos F. (2020). Insect responses to heat: Physiological mechanisms, evolution and ecological implications in a warming world. Biol. Rev..

[34] Fukuhara R., Kageyama T. (2013). Structure, gene expression, and evolution of primate copper chaperone for superoxide dismutase. Gene.

[35] Umasuthan N., Bathige S.D.N.K., Revathy K.S., Lee Y., Whang I., Choi C.Y., Park H.C., Lee J. (2012). A manganese superoxide dismutase (MnSOD) from *Ruditapes philippinarum*: Comparative structural- and expressional- analysis with copper/zinc superoxide dismutase (Cu/ZnSOD) and biochemical analysis of its antioxidant activities. Fish Shellfish Immun..

[36] Xikeranmu Z., Abdunasir M., Ma J., Tusong K., Liu X.N. (2019). Characterization of two copper/zinc superoxide dismutases (Cu/Zn-SODs) from the desert beetle *Microdera punctipennis* and their activities in protecting *E. coli* cells against cold. Cryobiology.

[37] Li D., Blasevich F., Theopold U., Schmidt O. (2003). Possible function of two insect phospholipid-hydroperoxide glutathione peroxidases. J. Insect Physiol..

[38] Kang Z.W., Liu F.H., Liu X., Yu W.B., Tan X.L., Zhang S.Z., Tian H.G., Liu T.X. (2017). The potential coordination of the heat-shock proteins and antioxidant enzyme genes of *Aphidius gifuensis* in response to thermal stress. Front. Physiol..

[39] An M.I., Choi C.Y. (2010). Activity of antioxidant enzymes and physiological responses in ark shell, *Scapharca broughtonii*, exposed to thermal and osmotic stress: Effects on hemolymph and biochemical parameters. Comp. Biochem. Physiol. B.

