# Supplementary material for: Decoding Peroxidase Gene Function in Heat Stress Adaptation of Tetranychus urticae: Unraveling Molecular Mechanisms of Short-Term Thermal Tolerance
Source: Antioxidants (Basel). 2025 May 8;14(5):562. doi: 10.3390/antiox14050562 (PMC12108298; doi:10.3390/antiox14050562)
Supplement: Supplementary file 1 [file antioxidants-14-00562-s001.zip › Table and Figure/Table S1.pdf]

Table S1. Primer sequences used in this study

Gene name	Primer name	Primer sequences (5’-3’)	Description
TuPOD1	*TuPOD1*-F	ATGATTAATTATTGTTTGATAGT	Primers for cloning
	TuPOD1-R	AAATACTTACAGACCATAATTA	
	q*TuPOD1*-F	GCACCTTTACCTTCAGCTCG	Primers for RT-qPCR
	q*TuPOD1*-R	TTCAGCTTGCGAAGGACAAC	
TuPOD2	*TuPOD2*-F	ACCTGTTGATTGCTGGAAAACA	Primers for cloning
	TuPOD2-R	CCATTGGTTTCTAGTTTGTCCGT	
	q*TuPOD2*-F	TCCAACATTCGACGAGGTGA	Primers for RT-qPCR
	q*TuPOD2*-R	ACGGAAGATCAGAACAGGCA	
TuPOD3	*TuPOD3*-F	TGGTTTTGTCAGTTGCTTTGGA	Primers for cloning
	TuPOD3-R	TGATTCAAACATTGTCTCACTCG	
	q*TuPOD3*-F	CCATCCGTCTTCCAGTCCAT	Primers for RT-qPCR
	q*TuPOD3*-R	AGCCATGATCACGACCTCTT	
TuPOD4	*TuPOD4*-F	ACAATCCCAATCATGAAGCTCA	Primers for cloning
	TuPOD4-R	GATTTCTGTTTCACTTGCGTCA	
	q*TuPOD4*-F	AGCCTTTACCTTCACCACGA	Primers for RT-qPCR
	q*TuPOD4*-R	TGAACACCTGATCCATGGCT	
TuPOD5	*TuPOD5*-F	GCCTTTCATCTGTCAACGCT	Primers for cloning
	TuPOD5-R	ACAAAACCTCGACTGGCTCA	
	q*TuPOD5*-F	GGTCACTCAATCCTTCCCGA	Primers for RT-qPCR
	q*TuPOD5*-R	ACTTCCGGGTCCATTCTGTT	
TuPOD6	*TuPOD6*-F	ATTGAGAGTTTAAAGGACAAT	Primers for cloning
	TuPOD6-R	TAATATCGCAACACTTTAAATG	
	q*TuPOD6*-F	GTGCAGCCTTCAGATTTGGT	Primers for RT-qPCR
	q*TuPOD6*-R	GGTGAGTCTTTGGGTCGGTA	
α-tubulin	q*α-tubulin*-F	TCTTGTCCCCTACCCTCGTA	Primers for RT-qPCR
	q*α-tubulin*-R	TTTCCATGTCGAGGGTCACA	
