# Supplementary material for: Decoding Peroxidase Gene Function in Heat Stress Adaptation of Tetranychus urticae: Unraveling Molecular Mechanisms of Short-Term Thermal Tolerance
Source: Antioxidants (Basel). 2025 May 8;14(5):562. doi: 10.3390/antiox14050562 (PMC12108298; doi:10.3390/antiox14050562)
Supplement: Supplementary file 1 [file antioxidants-14-00562-s001.zip › Table and Figure/Table S2-S5.pdf]

Table S2. Reaction system of PCR

Reagents	Volume (μl)
PrimeSTAR Max Premix (2X)	25 μl
Primer 1	1 μl
Primer 2	1 μl
Template	1 μl
RNase-free Water	5 μl

Table S3. Reaction conditions of PCR

Temperature	Time	Cycles
98 ℃	3 min	1
98 ℃	10 s	
55 ℃	15 s	35
72 ℃	30 s	
72 ℃	5 min	1
4 ℃	∞	

Table S4. Reaction system of RT-qPCR

Reagents	Volume (μl)
TB Green *Premix Ex Taq* II (Tli RNaseH Plus) (2X)	10 μl
PCR Forward Primer (10 μM)	0.8 μl
PCR Reverse Primer (10 μM)	0.8 μl
ROX Reference Dye II (50 X)	0.4 μl
DNA template	2 μl
RNase-free Water	6 μl

Table S5. Reaction conditions of RT-qPCR

Temperature	Time	Cycles
95 ℃	30 s	1
95 ℃	5 s	40
64 ℃	1 min	
95 ℃	15 s	1
60 ℃	1 min	
95 ℃	15 s	
