# Supplementary material for: Decoding Peroxidase Gene Function in Heat Stress Adaptation of Tetranychus urticae: Unraveling Molecular Mechanisms of Short-Term Thermal Tolerance
Source: Antioxidants (Basel). 2025 May 8;14(5):562. doi: 10.3390/antiox14050562 (PMC12108298; doi:10.3390/antiox14050562)
Supplement: Supplementary file 1 [file antioxidants-14-00562-s001.zip › Table and Figure/Table S6 and S7.pdf]

Table S6 Quality control table of RNA, cDNA, and plasmid used in cloning

Sample	Name	Density	D260/280
RNA	RNA-a	383.8	2.056
	RNA-b	352.6	2.014
	RNA-c	473.2	2.037
cDNA	DNA-a	529.1	1.994
	DNA-b	463.5	2.007
	DNA-c	576.9	2.041
Plasmid	Plasmid-a	937.4	2.033
	Plasmid-b	813.9	2.016
	Plasmid-c	1006.7	2.005

Table S7 Quality control table of RNA and cDNA used in RT-qPCR

Sample	Name	Density	D260/280
RNA	RNA-25℃-a	352.8	2.094
	RNA-25℃-b	343.6	2.035
	RNA-25℃-c	301.4	2.064
	RNA-36℃-a	371.8	2.007
	RNA-36℃-b	338.2	2.064
	RNA-36℃-c	385.1	2.018
	RNA-39℃-a	471.9	2.058
	RNA-39℃-b	294.7	2.042
	RNA-39℃-c	261.3	2.036
	RNA-42℃-a	497.1	1.994
	RNA-42℃-b	463.2	2.017
	RNA-42℃-c	386.4	2.049
cDNA	cDNA-25℃-a	453.2	2.009
	cDNA-25℃-b	369.4	2.004
	cDNA-25℃-c	368.2	2.043
	cDNA-36℃-a	545.5	2.011
	cDNA-36℃-b	574.3	2.033
	cDNA-36℃-c	341.3	2.019
	cDNA-39℃-a	439.7	2.041
	cDNA-39℃-b	468.2	2.062
	cDNA-39℃-c	493.7	2.083
	cDNA-42℃-a	541.3	1.993
	cDNA-42℃-b	554.1	1.997
	cDNA-42℃-c	517.0	2.034
