# Supplementary figures and images for: Decoding Peroxidase Gene Function in Heat Stress Adaptation of Tetranychus urticae: Unraveling Molecular Mechanisms of Short-Term Thermal Tolerance
Source: Antioxidants (Basel). 2025 May 8;14(5):562. doi: 10.3390/antiox14050562 (PMC12108298; doi:10.3390/antiox14050562)

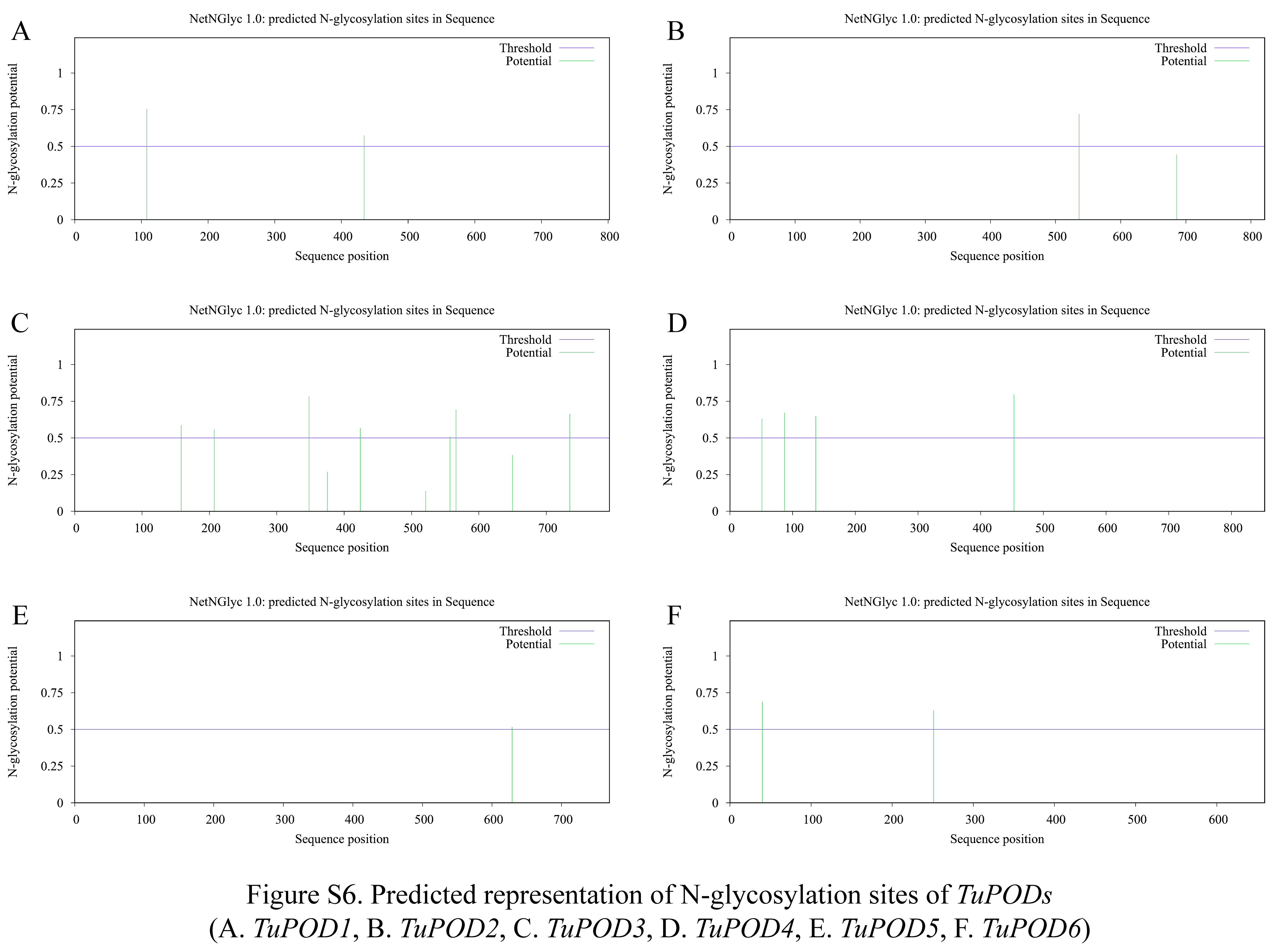

Supplement: Supplementary file 1 [file antioxidants-14-00562-s001.zip › Table and Figure/Figure S6.tif]

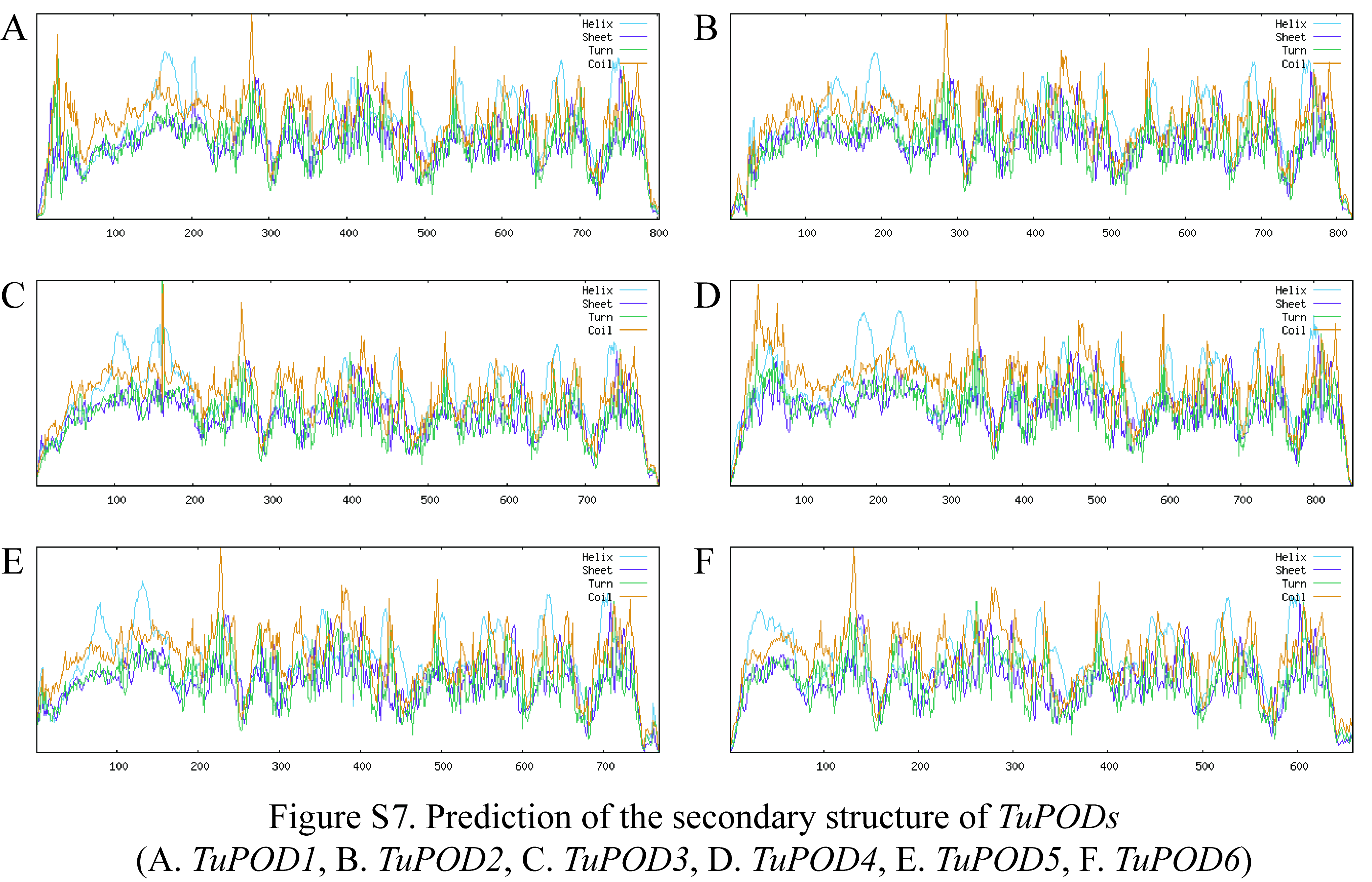

Supplement: Supplementary file 1 [file antioxidants-14-00562-s001.zip › Table and Figure/Figure S7.tif]
